# A Gamma Polarimeter for Neutron Polarization Measurement in a Liquid Deuterium Target for Parity Violation in Polarized Neutron Capture on Deuterium

**DOI:** 10.6028/jres.110.028

**Published:** 2005-06-01

**Authors:** A. Komives, A. K. Sint, M. Bowers, M. Snow

**Affiliations:** DePauw University, Greencastle, IN 46135; Indiana University, Bloomington, IN 47408

**Keywords:** deuterium, gamma polarimeter, neutron polarization, weak meson coupling constants

## Abstract

A measurement of the parity-violating gamma asymmetry in n-D capture would yield information on N-N parity violation independent of the n-p system. Since cold neutrons will depolarize in a liquid deuterium target in which the scattering cross section is much larger than the absorption cross section, it will be necessary to quantify the loss of polarization before capture. One way to do this is to use the large circular polarization of the gamma from n-D capture and analyze the circular polarization of the gamma in a gamma polarimeter. We describe the design of this polarimeter.

## 1. Introduction

The weak interaction is mediated by the W^+−^ and Z^0^ gauge bosons. Because these particles are very massive their range is too short to allow their direct exchange between nucleons interacting via the weak force. Instead the effects of the weak boson exchange are mediated through light mesons, which have the range to interact strongly with another nucleon. Because the weak boson range is so small, it can be taken to occur at a point allowing the interaction to be described by the exchange of a light meson with a weak coupling at one end and a strong coupling at the other. The weak meson coupling constants determine the strength of the weak interaction for a specific meson exchange.

In 1980, Desplanques, Donoghue and Holstein (DDH) calculated, from the Standard Model, theoretical values for the weak meson coupling constants shown in Table 1 [1] [2]. Since that time, no experiment has been able to measure these constants and the validity of the meson exchange model for weak interactions in the N-N system has yet to be established.

Currently there is an effort underway at the Los Alamos Neutron Scattering Center (LANSCE) to measure the pion coupling constant $H_{\pi }^{1}$ using transversely polarized neutrons on protons [3] [4] [5]. Measuring the directional asymmetry of the 2.2 MeV gammas from the newly formed deuterium nuclei will yield a value for $H_{\pi }^{1}$. With minimum modifications, this experimental setup can be used to measure the gamma directional asymmetry, *A_γ_*, from tritium nuclei produced by the capture of transversely polarized neutrons on deuterium. This asymmetry is related to the pion and rho isoscalar coupling constants via [6] [7]

$$A_{\gamma }=0.92H_{\pi }^{1}-0.5H_{\rho }^{0}-0.16H_{\omega }^{0}+0.10H_{\rho }^{1}-0.002H_{\omega }^{1}.$$ (1)

As with other low energy parity violating observables involving neutrons, this asymmetry is dominated by the isovector pion and isoscalar rho contributions if these couplings are close to the DDH “best” values. Such a measurement in the n-D system would yield information on N-N parity violation independent of the n-p system. The previous measurement obtained a result consistent with zero: *A_γ_* = (4.2 +/− 3.8) × 10^−6^ [8].

## 2. Neutron Depolarization in Deuterium

The expected size of *A_γ_* for d(n,*γ*)t is about 10 times larger than for p(n,*γ*)d due to the parity conserving part of the gamma decay, M1 transition, being suppressed relative to the PNC part, an E1 transition [6] [7]. Despite this, the probability of neutron depolarization in the deuterium target is a more serious problem than for p(n,*γ*)d. The scattering cross section on deuterons is about 10^3^ to 10^4^ times larger than the neutron capture cross section. The large number of scatters a particular neutron will experience before capture along with the fact the ground state of the deuterium molecule is orthodeuterium, having non-zero angular momentum, implies significant neutron depolarization in the target. Therefore to suppress depolarization due to multiple scattering in a pure deuterium target would require a thin, weakly-absorbing target of liquid orthodeuterium. The counting statistics required to see the parity violating effect then become comparable to that needed for p(n,*γ*)d. (The efficiency of the CsI gamma detector array for the 6.2 MeV gammas from nD capture is actually higher than for the 2.2 MeV gammas from np capture, so this is not an issue.) The types of systematic effects for this experiment are generally very similar to those for p(n,*γ*)d. However, some of the possible systematic effects for this experiment are larger relative to the parity violating signal due to the need to use a thin target of D_2_. In addition, the circular polarization of the M1 gamma ray is much larger, around 42 % [9], than in p(n,*γ*)d, around 0.3 %, and this can also lead to larger systematic effects. Therefore, the measurement of parity violation in d(n,*γ*)t is actually more difficult than in p(n,*γ*)d despite the expected order-of-magnitude larger size of the effect. Any measurement would also require an auxiliary determination of the neutron polarization upon capture in the target. This can be done by measuring the circular polarization of the gamma ray.

For less than 100 % neutron polarization it is important to note the relationship between the actual directional gamma asymmetry, *A_γ_*_,true_, and the observed directional gamma asymmetry, *A_γ_*_,obs_, is

$$A_{\gamma ,\text{true}}=\frac{A_{\gamma ,\text{obs}}}{P_{n}},$$ (2)

where *P*_n_ is the neutron polarization. Clearly, knowledge of the neutron depolarization is critical for a nonzero measurement of $H_{\rho }^{0}$.

Upon capture of transversely polarized neutrons by deuterium nuclei, the resulting 6.2 MeV gammas will be circularly polarized. The amount of circular polarization, *P_γ_*, is related to *P*_n_ by

$$P_{n}=\frac{P_{\gamma }}{R^{d}},$$ (3)

where *R*^d^ is the polarization parameter for the 6.2 MeV gammas, −0.42 +/− 0.03 [9]. So if *P_γ_* can be measured, then *P*_n_ for neutrons on a deuterium target can be determined.

## 3. Gamma Polarimeter Construction

In order to measure *P_γ_*, two transmission gamma polarimeters have been constructed. These devices use the gamma-electron spin dependent part of the Compton scattering cross section to filter out gammas of a particular polarization state. Each polarimeter is a solenoid with a Permendur, a copper-nickel-vanadium alloy, core. The magnetic field created by the solenoid polarizes a fraction of electron spins in the Permendur. As the gammas traverse the magnetized core, many will Compton scatter with electrons reducing the number of gammas exiting the polarimeter. The exact amount of reduction depends on the circular polarization of the gammas and the direction of the electron spins which is determined by the direction of the polarimeter’s magnetic field. If *S*_+_ and *S*_–_ are the number of gammas that pass through the core without being scattered with the magnetic field in the +(–) direction, then the asymmetry, *A*, will be

$$A=\frac{S_{+}-S_{-}}{S_{+}+S_{-}}=\eta P_{\gamma },$$ (4)

where *η* is the analyzing power of the polarimeter.

The analyzing power is primarily a function of polarimeter design,

$$\eta =n_{0}L\nu \sigma_{c},$$ (5)

where *n*_0_ is the number density of electrons in the core, *L* is the core length, *ν* is the fraction of electrons that are magnetized, and *σ*_c_ is the Compton scattering cross section due to the circular polarization of the gammas. In principle it is possible to calculate *η*; however, in practice it is more accurate to measure it using the actual polarimeter. To do this, a ^32^S target will be used. The resulting 5.44 MeV circularly polarized gammas from capture of polarized neutrons will pass through the polarimeter and the asymmetry *A* determined. This in turn is related to the analyzing power by

$$\eta =\frac{A}{P_{n}R^{s}},$$ (6)

where *R*^s^ is the polarization parameter of the 5.44 MeV gamma of ^32^S, 0.50 [9]. Because ^32^S is a spin 0 nucleus, the neutron will not flip its spin while in the target and *P*_n_ can simply be measured with a supermirror placed upstream of the target.

**Fig. 1:**
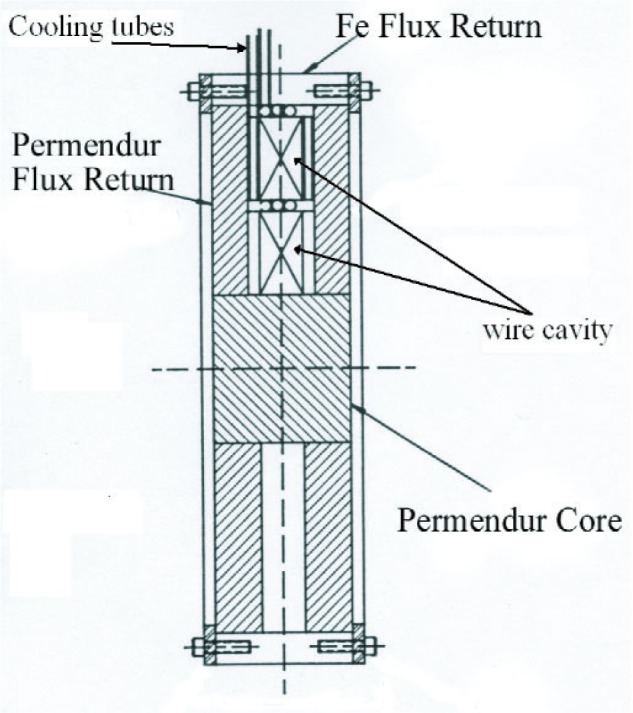
A cross sectional drawing of the cylindrical polarimeters built. The diameter is 24.4 cm and the length is 6.6 cm.

**Fig. 2:**
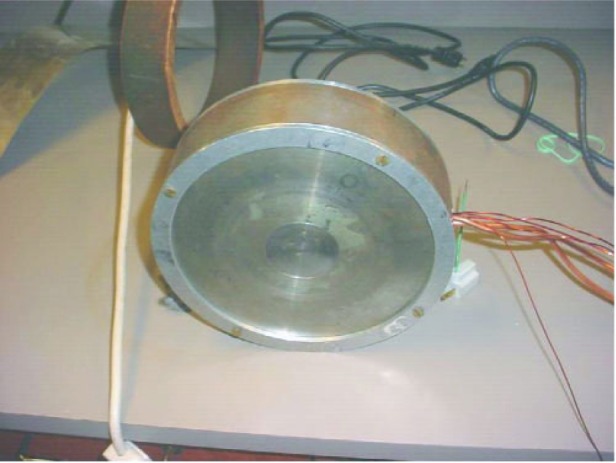
A photograph of a completely assembled polarimeter.

Figure 1 shows a cutaway drawing of the polarimeter while Fig. 2. shows a photo of a completely assembled polarimeter. Each polarimeter was wound with 495 turns of 16 AWG Super Hyslik^1^ 200 magnet wire. Halfway through the windings, three turns of 0.476 cm diameter copper tubing were wound and an additional three turns were added after the remainder of the wire was wound. Water flowing through the copper tubing keeps the insulation on the magnet wire from melting. Each polarimeter was designed to operate at 3000 amp-turns to produce a saturated magnetic field in the core of 2.5 Tesla. Wound around each polarimeter core are a few turns of 18 AWG wire that will be used to sense when the magnetic field changes.

## 4. Measurement Performance

Assuming 1.2 × 10^8^ polarized neutrons/cm^2^/sec are produced at the NG6 end station at the National Institute of Standards and Technology Center for Neutron Research [10], two 10 % relative efficiency Ge detectors and two polarimeters are used for a month of data collecting, *P_γ_* can be determined to 1 %. This will allow a 10 % measurement, most of the error being due to the error in *R*^d^, of *P*_n_ in the deuterium target.

## 5. Conclusion

We have built two transmission gamma ray polarimeters that will be used to measure the circular polarization of 6.2 MeV gammas from the capture of polarized neutrons on deuterium. This measurement will allow the determination of the neutron depolarization in deuterium, important for the measurement of the rho isoscalar coupling constant using polarized neutrons on deuterium.

## Figures and Tables

**Table 1:** Theoretical estimates of the weak meson coupling constants [1][2]

Exchanged meson	Isospin change	Coupling constant	Best value (×10^−6^)	Reasonable range (×10^−6^)
π	1	$H_{\pi }^{1}$	1.08	0.0:2.71
*ρ*	0	$H_{\rho }^{0}$	1.59	−1.59:4.29
*ρ*	1	$H_{\rho }^{1}$	0.03	0:0.053
*ρ*	2	$H_{\rho }^{2}$	1.33	−1.06:1.54
*ρ*	1	${H^{\prime }}_{\rho }^{1}$	0.00	None
*ω*	0	$H_{\omega }^{0}$	0.80	−2.39:4.29
*ω*	1	$H_{\omega }^{1}$	0.48	0.32:0.80

## Footnotes

1 Certain commercial equipment, instruments, or materials are identified in this paper to foster understanding. Such identification does not imply recommendation or endorsement by the National Institute of Standards and Technology, nor does it imply that the materials or equipment identified are necessarily the best available for the purpose.
